# Indolent Breast Lymphomas: A Case Series and Review of Literature

**DOI:** 10.4021/wjon681w

**Published:** 2013-07-15

**Authors:** Nauman Shahid, Kamal KS Abbi, Khurram Hameed, Iman Mohamed

**Affiliations:** a Department of Hematology and Oncology, University of Toledo Medical Center, Toledo, OH, USA

**Keywords:** Breast lymphoma, Marginal Zone Lymphoma, Small Lymphocytic Lymphoma

## Abstract

Indolent breast lymphomas (IBL) are a rare form of extranodal lymphoma. There are very few reported cases of IBL presenting as a breast mass. We report two cases of IBL, a Marginal Zone Lymphoma (MZL) and a Small Lymphocytic Lymphoma (SLL), which were discovered on routine mammogram and later confirmed by biopsy and flow cytometery. Patient with MZL underwent chemotherapy and radiation therapy whereas patient with SLL was treated with chemotherapy alone.

## Introduction

Hematopoietic malignancies can be encountered in the breast and can occasionally mimic primary breast cancers. Although lymphomas are generally considered as tumors of lymph nodes about 25-40% arise at extranodal sites [1, 2]. Breast lymphomas make up 1-2% of all extranodal lymphomas [1] and 0.04-0.5% of all breast malignancies [3]. Most of the breast lymphomas are high grade lymphomas. Because there are such few reported cases, that there is no general consensus on the incidence of IBL presenting as a breast mass. We present two cases of IBL which were discovered on routine screening mammograms. Issues in presentation, diagnosis and management will be discussed.

## Case Report

### Case 1

A 54-year-old woman presented to the oncology clinic after having an abnormal routine mammogram of her left breast which was read as BIRADS category 0. Therefore, the patient underwent a digital mammogram which was read as BIRADS category 4 and revealed a mass at 7 o’clock position, consistent with an abnormal intramammary lymph node. Subsequently, the patient had an ultrasound and an ultrasound guided core biopsy which showed findings consistent with atypical lymphoid infiltrate however, a lymphoproliferative disease could not be ruled out. The patient was referred to the breast surgery clinic where the patient underwent an excisional biopsy. The pathology report of the mass came back as a low grade lymphoma consistent with marginal zone lymphoma which was positive for CD 3, CD 20 and CD 43 and negative for CD 10. PET-CT scan showed a right infrahilar enlarged lymph node as well. The patient refused to have a bone marrow biopsy done. LDH and hgb were 146 IU/L and 13.5 g/dL, respectively, both of which were within the laboratory reference range. Viral hepatitis panel was also negative. The patient was staged as stage IIE marginal zone lymphoma. The patient received 6 cycles of R-CHOP which was followed by external beam radiation and is currently on maintenance rituximab for 2 years. The patient’s repeat PET-CT scan showed resolution of mediastinal lymphadenopathy suggesting satisfactory response to the treatment.

### Case 2

A 56-year-old woman was referred to a breast surgery clinic after she was found to have an abnormal screening mammogram which was read as BIRADS category 0. The mammogram findings were confirmed by an ultrasound which showed a mass. The patient’s physical exam also revealed enlarged lymph nodes in the neck region. Her blood workup showed hgb of 11.8 gm/dl, platelet count of 244,000 and white cell count of 64,000 with 63% lymphocytes and 30% neutrophils. The patient underwent a right breast and cervical lymph node excisional biopsy. The pathology report came back as small lymphocytic lymphoma which was positive for CD 5, CD 23 and weakly positive for CD 20. CT scan of chest, abdomen and pelvis showed hilar, cervical and groin lymphadenopathy. The patient was referred to the medical oncologist where she was started on Chlorambucil but the response was not satisfactory with white cell count worsening to 130,000, therefore, the chemotherapy was switched to Fludarabine, Cytoxan and Rituximab. She received 6 cycles of FCR. The patient’s PET-CT scan after the chemotherapy did not show lymphadenopathy and her white cell count improved to 25,000 with 79% lymphocytes. The patient continued to have serial CBC done which again showed increasing WBC and decreasing Hgb and platelet count. After being in remission for approximately 30 months chemotherapy was reinitiated due to the relapse.

## Discussion

Primary breast lymphomas are a rare form of localized extranodal lymphoma whereas secondary involvement of the breast is relatively more common. The small amount of lymphoid tissue in the breast could account for the rarity of breast lymphoma when compared with other organs [4]. B-cell lymphoma occurs more frequently than T-cell lymphoma in the breast [5] and interestingly right breast is more frequently involved than the left breast [6]. Most breast lymphomas are high-grade malignant neoplasms, mainly large cell and Burkitt type. Indolent lymphomas of the breast have been exceedingly rare.

In 1972 Liao and Wiseman defined Primary Breast Lymphoma (PBL) as only tumors that are stage IE (lymphoma limited to the breast) and stage IIE (lymphoma limited to the breast and axillary lymph nodes), excluding those tumors that may have originated at non-breast sites [7]. In our case series we report two cases of IBL, which were stage IIE and stage IIIE at diagnosis.

The largest series investigating clinical outcomes in breast lymphomas of indolent histology was published by International Extranodal Lymphoma Study Group (IELSG) in 2009 which provides perhaps one of the best insight to date into the clinical behavior of these rare diseases [8].

Both our cases had Low Grade Non Hodgkin Lymphoma (NHL), MZL and SLL. There is very little known about the pathogenesis of histologically ‘low-grade’ breast lymphomas. A pathophysiologic role of chronic infections has been identified in some extranodal NHL such as gastric (Helicobacter pylori), ocular adnexal (Chlamydia psittaci) and skin (Borrelia burgdorferi) MZL [9], but such correlations have not been reported in the literature regarding breast lymphoma.

There are no characteristic clinical features of Indolent Breast Lymphomas. They most frequently present as a mass that may mimic carcinoma [10]. Occasionally the mass may be painful [10] or there may be skin fixation. Skin involvement is seen with diffused parenchymal involvement, which is more common in high grade lymphoma.

Diagnosis can be made by ultrasound guided biopsy. Fine needle aspiration with flow cytometry can provide the diagnosis. Core biopsy is superior for subtyping of lymphomas.

Due to the scarcity of reported cases of IBP, no pathognomonic imaging features have been identified and do not help in differentiating from breast cancer in patients with lymphomas. Radiographically, indolent breast lymphomas appear as non-specific circumscribed masses that lack calcification [11]. The ultra sound findings of IBL are variable and they can appear as well or ill-defined, hyper or hypo echoic, focal or diffuse and with or without skin thickening [11]. The MRI appearance of breast lymphoma appears as ill-defined non-speculative hypointense mass with rapid and strong enhancement on T1 weighted images [12]. Hybrid PET/CT has become the standard imaging modality for staging, follow up and treatment response in patients with lymphoma [13]. Breast lymphoma is staged according to the Ann Arbor staging system.

In general, treatment of IBL follows treatment recommendations for lymphomas of the same stage and histology in other locations. Treatment options include surgery, chemotherapy and radiation therapy. The choice of treatment regimen should be based upon histologic subtype, disease extent, and the individual patient. Stage IE indolent histology can be treated with local radiation alone rather than chemotherapy but patients with certain tumor characteristics may require special modification to this approach [8]. In addition radiation may not be the preferred therapy for the very young patient where the long term risk of breast cancer is a concern. Stage IIE and advanced disease is generally treated with systemic chemotherapy, monotherapy, or observation, according to patient symptoms. Radiation therapy can be used to provide effective local control or may be adjuvant to chemotherapy. Surgical treatment ranging from simple to radical mastectomy is often not indicated [14].

IBL is a rare entity in the spectrum of malignant breast disease. There are at present no clear clinical or radiological features that distinguish it from any other type of infiltrating breast carcinoma. It should be considered in the differential of breast masses lacking features of speculation and microcalcification. Adequate tissue biopsy for histological evaluation and immunophenotyping is essential. IBL is not a surgical disease and can be treated successfully with combined chemotherapy and radiation therapy.
